# Can deep learning reduce the time and effort required for manual segmentation in 3D reconstruction of MRI in rotator cuff tears?

**DOI:** 10.1371/journal.pone.0274075

**Published:** 2022-10-10

**Authors:** Hyojune Kim, Keewon Shin, Hoyeon Kim, Eui-sup Lee, Seok Won Chung, Kyoung Hwan Koh, Namkug Kim

**Affiliations:** 1 Department of Orthopedic Surgery, Daejeon Eulji Medical Center, Eulji University School of Medicine, Daejeon, Republic of Korea; 2 Department of Convergence Medicine, Asan Medical Institute of Convergence Science and Technology, Asan Medical Center, University of Ulsan College of Medicine, Seoul, Republic of Korea; 3 Department of Orthopedic Surgery, Asan Medical Center, College of Medicine, University of Ulsan College of Medicine, Seoul, Republic of Korea; 4 Department of Orthopaedic Surgery, Konkuk University School of Medicine, Seoul, Republic of Korea; Korea National University of Transportation, REPUBLIC OF KOREA

## Abstract

**Background/Purpose:**

The use of MRI as a diagnostic tool has gained popularity in the field of orthopedics. Although 3-dimensional (3D) MRI offers more intuitive visualization and can better facilitate treatment planning than 2-dimensional (2D) MRI, manual segmentation for 3D visualization is time-consuming and lacks reproducibility. Recent advancements in deep learning may provide a solution to this problem through the process of automatic segmentation. The purpose of this study was to develop automated semantic segmentation on 2D MRI images of rotator cuff tears by using a convolutional neural network to visualize 3D models of related anatomic structures.

**Methods:**

MRI scans from 56 patients with rotator cuff tears (T2 Linear Coronal MRI; 3.0T, 512 mm × 512 mm, and 2.5-mm slice thickness) were collected. Segmentation masks for the cuff tendon, muscle, bone, and cartilage were obtained by four orthopedic shoulder surgeons, and these data were revised by a shoulder surgeon with more than 20 years’ experience. We performed 2D and 3D segmentation using nnU-Net with secondary labels for reducing false positives. Final validation was performed in an external T2 MRI dataset (10 cases) acquired from other institutions. The Dice Similarity Coefficient (DSC) was used to validate segmentation quality.

**Results:**

The use of 3D nnU-Net with secondary labels to reduce false positives achieved satisfactory results, even with a limited amount of data. The DSCs (mean ± SD) of the cuff tendon, muscle, bone, and cartilage in the internal test set were 80.7% ± 9.7%, 85.8% ± 8.6%, 97.8% ± 0.6%, and 80.8% ± 15.1%, respectively. In external validation, the DSC of the tendon segmentation was 82.74±5.2%.

**Conclusion:**

Automated segmentation using 3D U-Net produced acceptable accuracy and reproducibility. This method could provide rapid, intuitive visualization that can significantly facilitate the diagnosis and treatment planning in patients with rotator cuff tears.

## Introduction

A rotator cuff tear (RCT) is a common source of shoulder pain and disability. In combination with physical examinations and clinical history, imaging evaluations play a pivotal role in diagnosing RCTs. The imaging modality most commonly used in RCT detection is magnetic resonance imaging (MRI) [1, 2]. The primary reason for performing MRI in cases of suspected RCT is to determine the extent of the tear and help orthopedic surgeons in deciding whether rotator cuff repair should be performed. This explains why MRI reports include descriptions of important RCT characteristics, such as location, degree of tendon involvement, partial or full thickness, size, and degree of retraction. However, while the tear pattern is not often reported in MRI readings, it plays a significant role in RCT management, especially in surgical treatment. In comparison with radiologists’ reports of knee MRI, which regularly includes descriptions of the shape of a meniscal tear, the rotator cuff tendon tear pattern remains relatively challenging to elucidate through two-dimensional (2D) MRI [3].

The rotator cuff tear pattern can play an important role in the surgeon’s approach and selection of repair procedures as well as the likelihood of clinical success after repair [4–6]. The availability of this information before surgery can be useful for the surgeon and will permit complete surgical planning, allowing the surgeon to provide prognostic information to patients based on surgical success rates for different tear patterns. Accurate determination of the tear pattern can also help in deciding whether the tear is repairable. In addition, a correctly defined tear pattern can help determine if there is enough tendon tissue remaining to allow marginal convergence during the repair. Previous studies demonstrated that the characterization of RCTs and their patterns by MRI could be difficult before arthroscopic examination [7, 8]. Even though a classification system for RCT patterns using 2D MRI has been presented in the literature, this system is not as accurate as that seen during arthroscopy [5, 9].

Gyftopoulos et al. tried to demonstrate that 3D visualizations of images of the rotator cuff can improve the accuracy of characterizing RCT patterns in comparison with 2D MRI-based techniques [10]. Although 3D visualized images can offer more intuitive visualization and better facilitate treatment planning than 2D MRI, the currently used method of manual segmentation is time-consuming and lacks reproducibility. 3D reconstruction of cuff tendon MRI requires high-quality images (thin section, 1-mm slice), a specialist with extensive musculoskeletal training, and over 4–5 hours of manual tasks. For these reasons, 3D reconstruction of cuff tendons by using MRI has not gained popularity in clinical practice.

In this regard, recent advancements in deep learning technology may provide a solution through automatic segmentation of 3D images to produce 3D reconstructed images efficiently and accurately. Trials and techniques for this approach have been proposed, and its usefulness has been proven for images of the rotator cuff tendon [11–14]. Kim et al. investigated a deep learning method that can rule out a substantial rotator cuff injury in individuals with suspected rotator cuff tear by using conventional shoulder radiographs [12]. Although their method showed a high level of sensitivity, it was insufficient to identify various patterns of tendon tears due to the use of X-ray images. G Medina et al. demonstrated the usefulness of segmentation of the supraspinatus, infraspinatus, and susbscapularis in MRI using deep learning [13]. However, the presence and shape of the rotator cuff tendon was difficult to evaluate in the segmentation result since the authors did not focus on tendon segmentation. Shim et al. proposed a 3D convolutional neural network (CNN)-based method for diagnosing the existence or absence of an RCT, classifying tear size, and visualizing the tear location in 3D [14]. This method can facilitate estimation of the size and position of a tear, but it shows the limitation of not being able to display the detailed tear patterns.

However, the techniques and usefulness of automatic 3D visualization of the whole structure of the rotator cuff tendons has not been well established and proven yet. Unlike the signal of bone or cartilage tissue, which can be easily differentiated from other tissue, such as cuff tendon, biceps tendon, reliable and reproducible segmentation for rotator cuff tendon can easily lead to errors. To improve the quality of segmentation efficiency, a proper understanding of the anatomical structures’ relevance is important. We assumed that this problem could be resolved by using a protocol based on 3D U-Net instead of 2D U-Net, which allows consideration of the images’ relationships. In addition, construction of secondary labels for the biceps tendon, which is named different from cuff tendon, can enhance the quality of automated segmentation. Thus, the purpose of this study was to develop automated semantic segmentation on 2D MRI images of the rotator cuff tendon by using a CNN to visualize 2-dimensional images of cuff tissue into three dimensions. We hypothesized that our constructed segmentation protocol for 3-dimensional visualization could consistently yield good accuracy and reproducibility in internal and external validation.

## Materials and methods

### Imaging datasets

This study was conducted after receiving approval from the institutional review board (AMC, No 2019–1026). The informed consent was not obtained because the collected data were analyzed anonymousl. Records from the MRI database of a single center were collected, retrospectively. The 2D MRI examinations were performed from January 2019 to February 2020 for patients with suspected RCTs in the shoulder. Among them, we excluded patients with (1) a surgical history for the shoulder joints, (2) suspicion of infection, and (3) traumatic fracture or deformity of the shoulder joint. After excluding these patients, a total of 56 MRI images were eligible and enrolled for this study. The mean patient age in the retained group was 63.7 years (male/female ratio, 24/32 patients; Table 1). All patients underwent an identical MRI protocol using the 3.0-T scanner (Ingenia).

**Table 1:** Demographic data.

	Demographic data
Total	56 MRI images
Sex	
Male (%)	24 (42.9)
Female (%)	32 (57.1)
Right/Left (%)	32 (57.1)/24 (42.9)
Age (year)	63.7 ± 9.3
Tear size (intact/partial/small/medium/large/massive)	10/6/6/14/12/8
SSP or ISP tear	51
SSc tear	32
Goutallier stage (0/1/2/3/4)	3/21/21/7/4

ISP, infraspinatus; SSc, subscapularis; SSP, supraspinatus

### Gold standard

**Fig 1:**
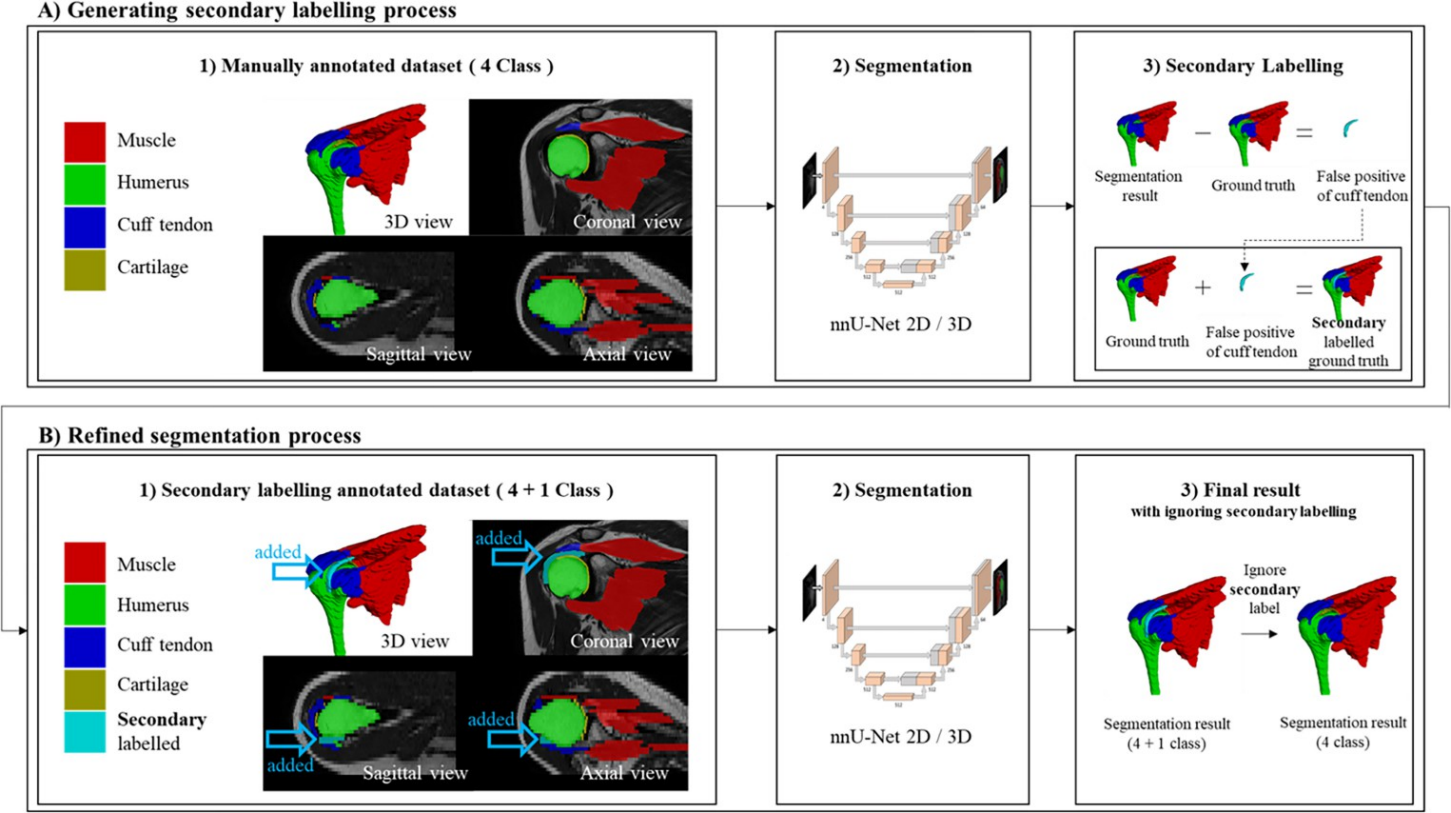
Study overview to improve 2D MRI visualization of rotator cuff tendons using segmentation network. In this study, training segmentation was conducted twice in order to improve the segmentation result of the cuff tendons, with A) secondary labelling process and B) refined segmentation training. In the secondary labelling process, a secondary labelled dataset was constructed based on the false-positive segmentation results for the cuff tendon of the segmentation model using the manually annotated dataset (4 classes; muscle, humerus, cuff tendon, cartilage), and the newly formed 4 + 1 class dataset was trained once more to obtain a refined segmentation result.

For the gold standard examination, masks for the cuff tendon, muscle, bone, and cartilage were manually segmented by four board-certified orthopedic shoulder surgeons, and the data was revised by one shoulder surgeon (KHK) with more than 20 years’ experience to ensure uniformity of the gold standard. Fig 1A shows an example of the gold standard masks. The labels were divided into four categories: muscle, humerus, cuff tendon and humeral head articular cartilage. The mean time required for manual segmentation in each case was 40 min.

### Overview

Our proposed segmentation learning process enhanced segmentation performance by efficiently removing irrelevant false positives. We introduced two training processes for this purpose.

The first process involved generating secondary labels as shown in Fig 1A. We trained a deep learning model using a manually annotated dataset (4 classes: muscle, humerus, cuff tendon, cartilage). Then, we computed false positives by comparing the ground truth with the prediction results. Lastly, we merged false positives with the ground truth to create the secondary labels shown as Fig 1A3. In this process, the four existing ground truth labels were preserved, and the false-positive part of the predicted results for the cuff tendon, the target, was added as the fifth label.

The second step was the refined segmentation process. The segmentation model was trained once more with the dataset to which secondary labelling was applied, and the fifth label added during secondary labelling was excluded from the prediction results, as shown in Fig 1B.

Fig 1 summarizes our study overview to improve 2D MRI visualization of rotator cuff tendons using 2D/3D U-Net.

### Deep learning protocol

**Fig 2:**
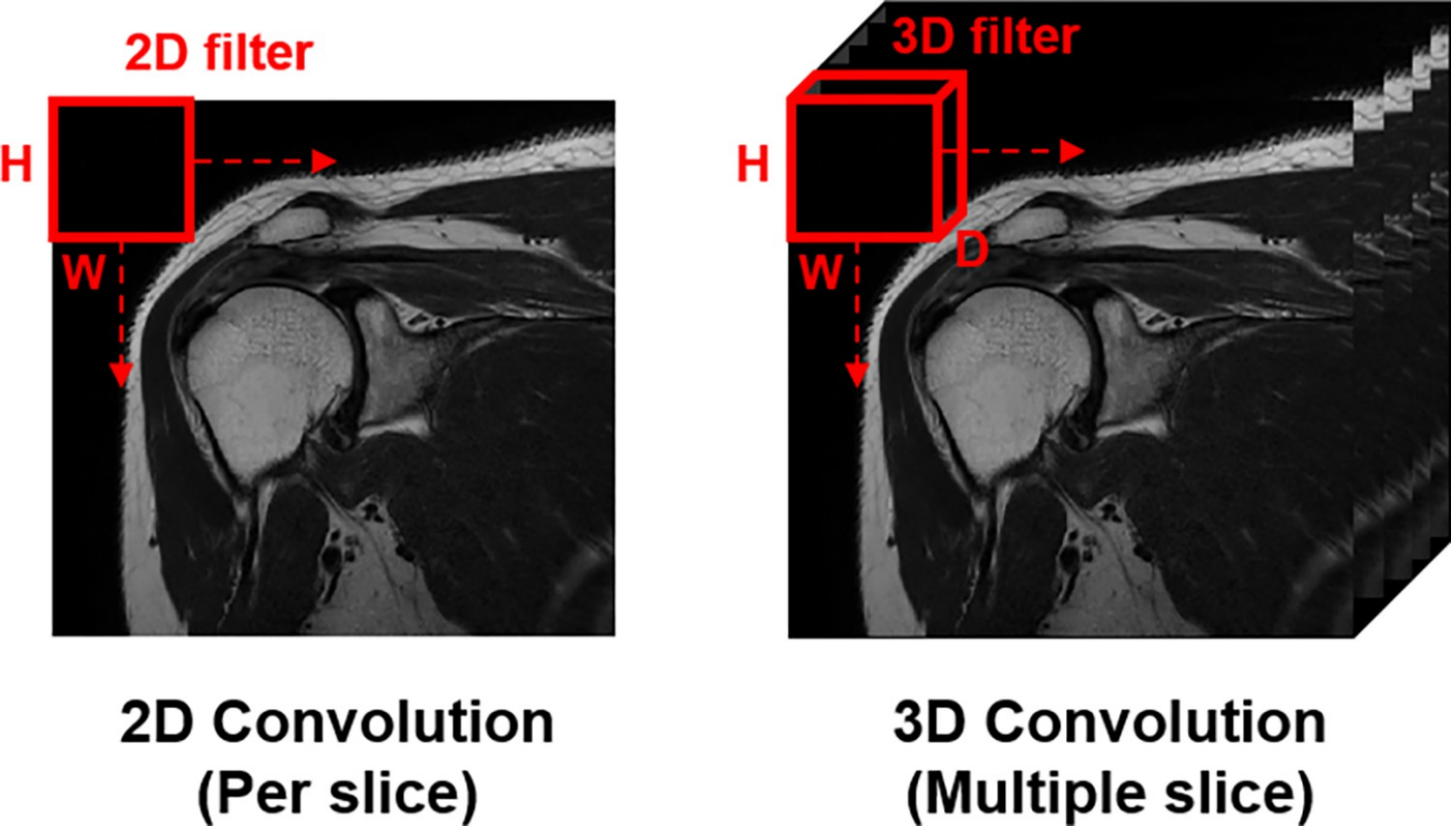
Comparison between 2D and 3D convolutional calculation. The number of dimensions represents the number of directions in the filter.

The U-Net is the deep learning model proposed by Olaf Ronneberger et al. for biomedical image segmentation [15]. The U-Net network consists primarily of two paths. The initial path is an encoder path, which compresses data using convolutional and max-pooling layer stacks. The second path is the symmetric expanding path used to enable precise localization with transposed convolutions. Thus, U-net is a highly advanced end-to-end segmentation model that can segment the image by pixel by combining this two-path information. We employed the Non new U-Net (nnU-Net) framework, which is optimized for 3D medical image organ segmentation on the basis of various intensities and spacing in computed tomography and MRI based on this U-Net architecture (official code: https://github.com/MIC-DKFZ/nnUNet) [16]. The nnU-Net is a patch-based learning model that shows high segmentation performance, especially in anisotropic medical images. In addition, nnU-Net provides the 2D nnU-Net (2D U-Net) and the full-resolution 3D nnU-Net (3D U-Net) based algorithm with advanced post-processing capabilities. In this application, both 2D U-Net and 3D U-Net were tested. The differences between 2D U-Net and 3D U-Net analysis methods are summarized in Fig 2. The performance of the anisotropic image varies depending on the 2D U-Net and 3D U-Net analysis methods, since the amount of image information varies according to the direction. Generally, 2D U-Net analyses images per slice, can be computed efficiently in memory, and is advantageous due to its relatively large dataset. In addition, Isensee et al. argued that 2D U-Net shows better segmentation performance than 3D U-Net in anisotropic images. However, understanding anatomical structures is inferior to 3D U-Net since it is analyzed by slices.

**Fig 3:**
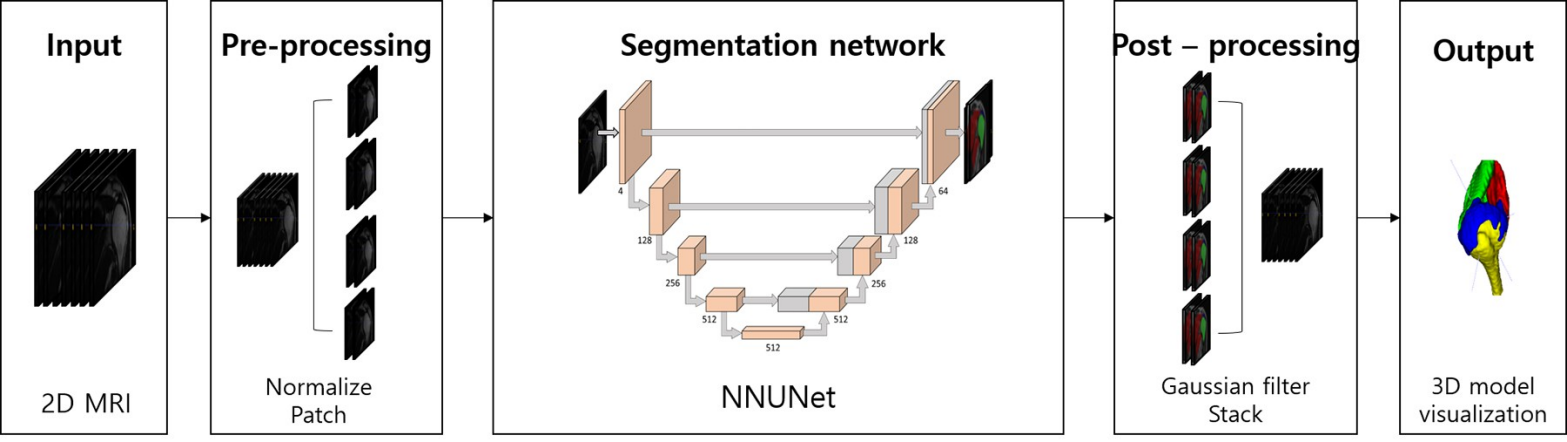
Pipeline of the nnU-Net segmentation model of 2D rotator cuff MRI.

The dataset was randomly divided into a training set (n = 34, 60%), a tuning set (n = 11, 20%), and a validation set (n = 11, 20%). The dataset was pre-processed by resampling all cases to a standard voxel spacing (2.5 mm × 0.29 mm × 0.29 mm) and a histogram-based normalization technique for each MRI image. During training, images of different sizes were input to the 2D U-Net and 3D U-Net. For the 2D U-Net, the input was 512 × 512, which corresponds to the full image resolution. For the 3D U-Net, a patch of size 16 × 320 × 320 became the input of the artificial intelligence model. Fig 3 illustrates the overall pipeline of 3D rendering of a rotating musculoskeletal system using the deep learning architecture of U-Net.

Since our research goal was to confirm the tear pattern of the tendon, we studied the relationship of labels that can best express the shape of the tendon. Tendons and muscles have ambiguous boundaries. Therefore, the tendon segmentation performance will depend on the relationship between labels. We used stochastic gradient descent for training with an early stopping option to prevent model overfitting. We set the batch size to 8, epoch size to 1000, and the loss function for learning was a combination of the Dice similarity coefficient (DSC) and weighted cross-entropy. Eq 1. depicted the loss function, where ε is the factor used to avoid division by zero. The model was implemented using Pytorch 1.5 in the Python 3.5 environment. We used a single GPU (Nvidia RTX—24 GB RAM) to train our model. For 2D U-Net and 3D U-Net, the training time on the dataset was 10 and 16 h, respectively. We applied extensive data augmentation such as rotations, scaling, brightness, contrast, and gamma augmentations to all training patches during the training.


$$Loss=-\sum_{i=0}^{n}\left[y_{i}\;\log\left(\hat{y_{i}}\right)+{(1-y}_{i})\;\log\left(1-\hat{y_{i}}\right)\right]+1-\frac{2|y_{i}\cap \hat{y_{i}}|+\varepsilon }{|y_{i}|+|\hat{y_{i}}|+\varepsilon }$$ Eq 1

### Secondary labelling

**Fig 4:**
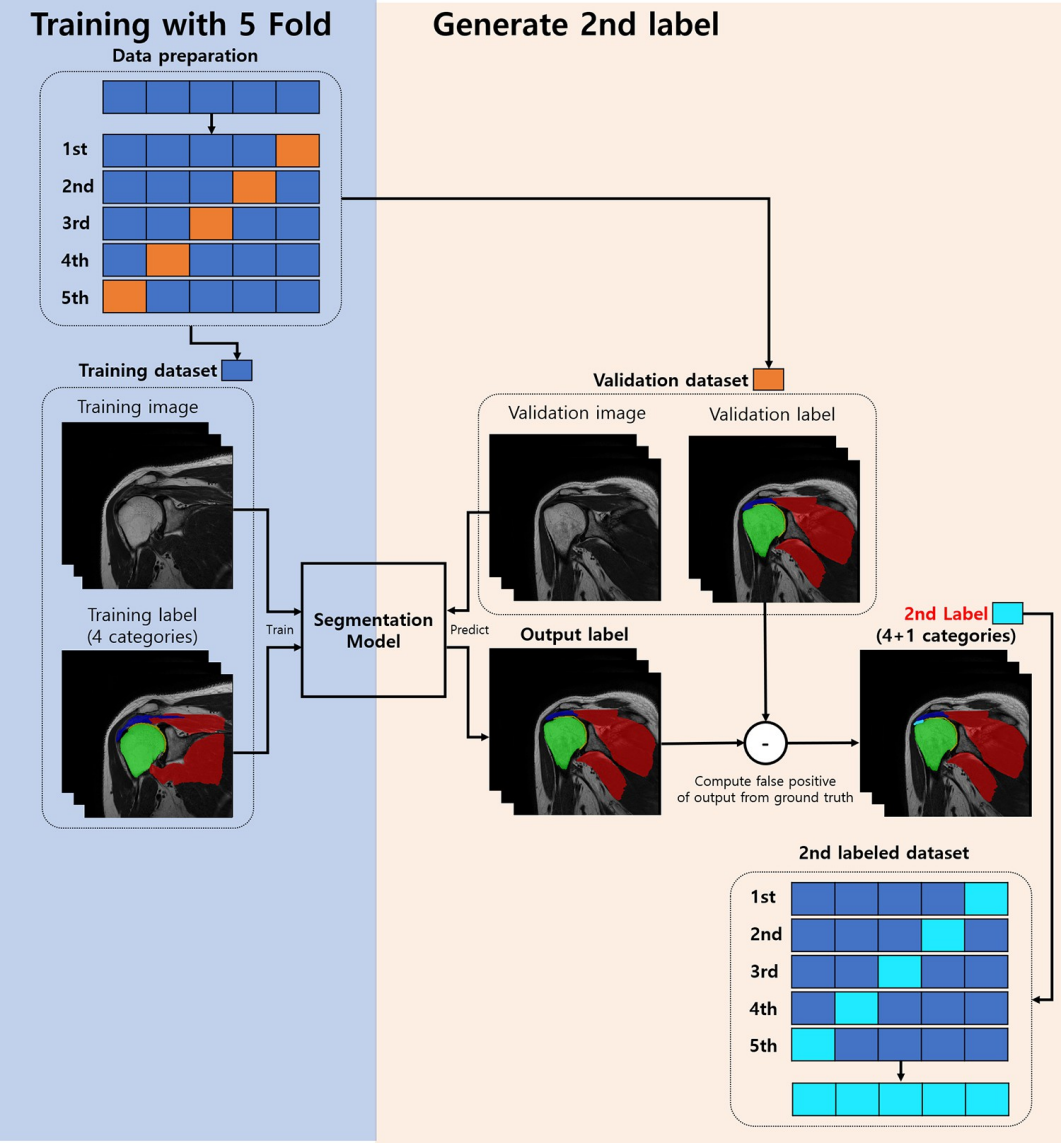
Process of adapting secondary labelling. To generate a complete secondarily-labelled data set (4+1 class), we split the data set into 5 folds and used 5 segmentation models trained by each fold.

Fig 4 depicts our strategy to enhance cuff tendon segmentation using secondary labeling. We observed that the biceps long head (BLH) tendon was predicted as a cuff tendon during our tests. However, since the BLH tendon was not labelled in the ground truth mask, false positives were removed using the secondary labeling technique. Inspired by Zhang et al., we assigned the BLH as the fifth category using the difference between the ground truth and the results of the first trained segmentation model [17].

To apply secondary labeling, labels must be reprocessed through entire datasets; thus, 5-fold cross-validation was performed to generate the secondary labeling. Fig 4 shows the process of generating the secondary labeling. First, the dataset was divided into 5-folds, and five training sessions were performed. For each fold, predictions were made using a validation dataset and compared with the ground truth, and false positives estimated to be biceps were designated as a new label (sky blue). Finally, the entire secondary labelled dataset was constructed using secondary label data from each of the five folds. When the dataset, including the secondary label dataset, was constructed in this way, a segmentation model was trained into the existing 4–5 classes, and the newly added class from the result was discarded.

### Evaluation metrics

In this study, the DSC and intersection over union (IoU) of the tendon cuff were used as the primary metrics to express the accuracy of the tear pattern of tendons. The DSC metric is widely used for performance evaluation of medical image segmentation tasks. The DSC is defined as the number of overlapped volumes between the segmentation result and the gold standard. In addition, IoU is a method for calculating the percent overlap between the target mask and our prediction output. The definitions and more detailed explanations of DSC and IoU metrics are well summarized in the study conducted by Taha & Hanbury [18].


$$\begin{array}{c} DSC=\frac{2∙TP}{2∙TP+FP+FN},\;IoU=\frac{TP}{TP+FP+FN}\\ {}*\;\mathrm{TP}=\mathrm{True}\;\mathrm{positive},\;\mathrm{FP}=\mathrm{False}\;\mathrm{positive},\;\mathrm{FN}=\mathrm{False}\;\mathrm{negative} \end{array}$$ Eq 2

Applying the DSC and IoU as an evaluation reference, intraobserver validation was performed to determine the most accurate and reproducible protocol. First, by comparing the 2D U-Net and 3D U-Net models, the segmentation metrics of the images were evaluated. Subsequently, evaluations based on the secondary label were conducted. To confirm the effect of the tear size on the segmentation metrics of reconstructed images, the segmentation performance according to the tendon cuff’s tear size was compared with the data derived from the model.

### External validation set

To confirm the robustness of segmentation, datasets were acquired from another independent center. Ten additional cases were selected for every different tear size and segmented manually by one shoulder surgeon who confirmed the intraobserver validation dataset. The manually segmented tendons were compared with the automatically reconstructed image by our study’s protocol. The external dataset was composed of equipment from various manufacturers, such as Siemens and Philips, and the strength of the magnetic field also varied (1.5T and 3T). Among these, we selected only the image produced by the coronal T2 protocol.

### Statistical analysis

The paired t-test was used to determine whether the difference in performance for each test was significant when the secondary label was adapted. One-way analysis of variance was performed to evaluate the performance of the artificial intelligence model according to the degree of tear of the patient’s cuff tendon. Both statistical analyses were performed using R version 3.5 (R Foundation for Statistical Computing, Vienna, Austria), and significance was set at a p-value of <0.05.

## Results

### Intraobserver validation

**Fig 5:**
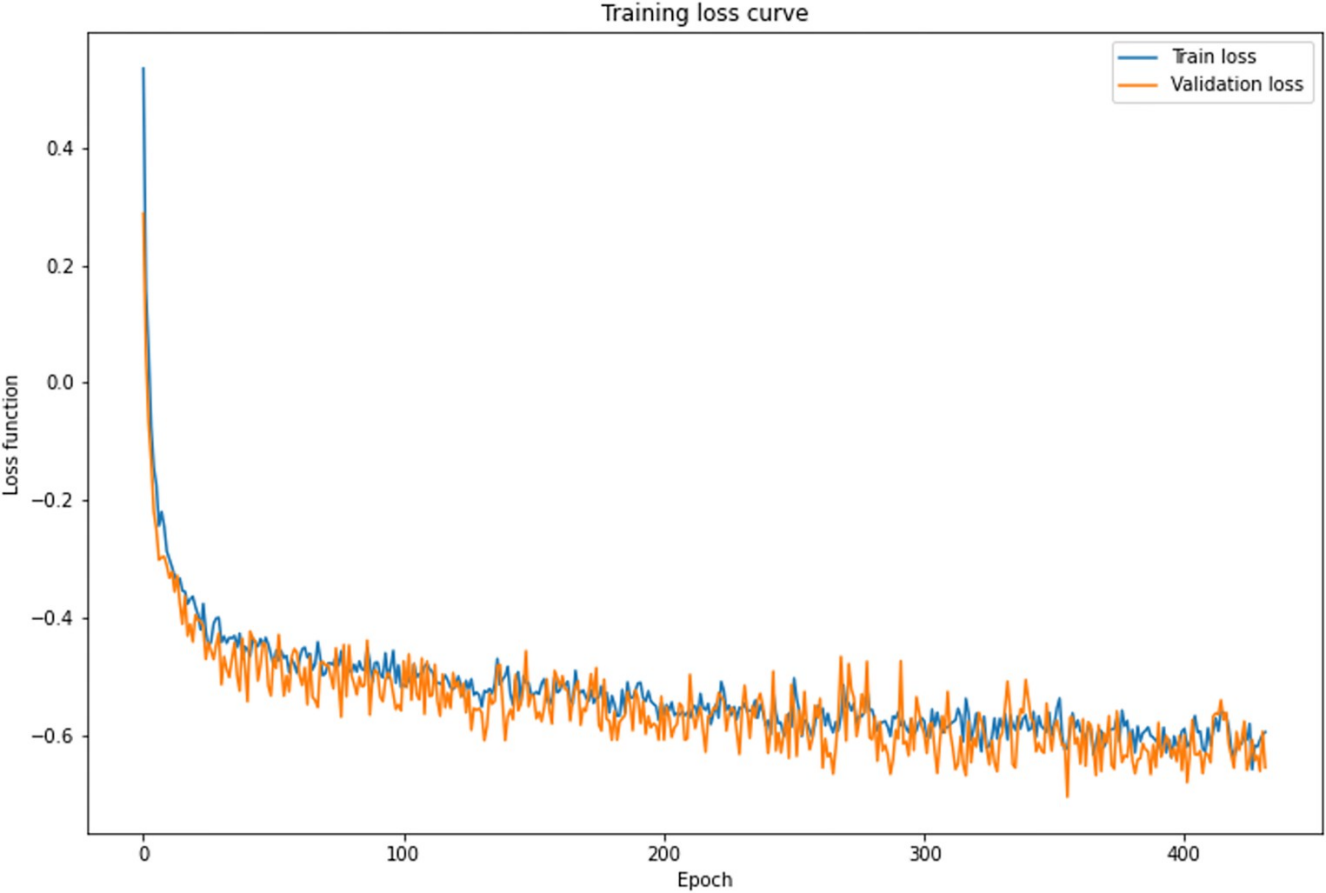
Training and validation loss curve of 3D semantic segmentation of the rotator cuff.

After the training was complete, DSC and IoU scores for the rotator cuff were computed with a gold standard mask. Fig 5 shows one of the training and validation curves in our study. Table 2 represents the evaluation results of our study. The highest DSC and IoU values were obtained when the secondary label was applied in the 3D U-Net based model. Using the 3D U-Net model with the secondary label, the tendon’s average DSC increased by 0.072. However, application of the secondary label in the 2D U-Net showed similar results within the error range.

**Table 2:** Segmentation result for each label in 2D and 3D U-Net according to the secondary labelling effect.

Model type (Number of parameters)	Secondary label		Muscle			Humerus			Tendon			Cartilage		Overall		
		DSC	IoU	p-value	DSC	IoU	p-value	DSC	IoU	p-value	DSC	IoU	p-value	DSC		IoU
2D	×	0.786 ±0.090	0.652 ±0.109	0.79	0.976 ±0.005	0.951 ±0.010	0.55	0.725 ±0.079	0.591 ±0.103	0.21	0.738 ±0.118	0.606 ±0.126	0.14	0.808 ±0.061 -		0.700 ±0.087
	O	0.786 ±0.099	0.653 ±0.110		0.976 ±0.006	0.950 ±0.011		0.728 ±0.080	0.596 ±0.102		0.738 ±0.124	0.605 ±0.135		0.810 ±0.063		0.701 ±0.090
3D	×	0.791 ±0.098	0.657 ±0.109	0.56	0.977 ±0.005	0.950 ±0.010	0.83	0.729 ±0.084	0.601 ±0.110	**0.04** *	0.745 ±0.121	0.625 ±0.093	0.78	0.812 ±0.065	-	0.708 ±0.090
	O	**0.797** **±0.095**	**0.665** **±0.112**		**0.978** **±0.006**	**0.953** **±0.012**		**0.801** **±0.094**	**0.651** **±0.117**		0.743 ±0.114	0.615 ±0.095		**0.830** **±0.073**		**0.727** **±0.092**

DSC, Dice similarity coefficient, IoU, intersection over union

*, p < 0.05

**Fig 6:**
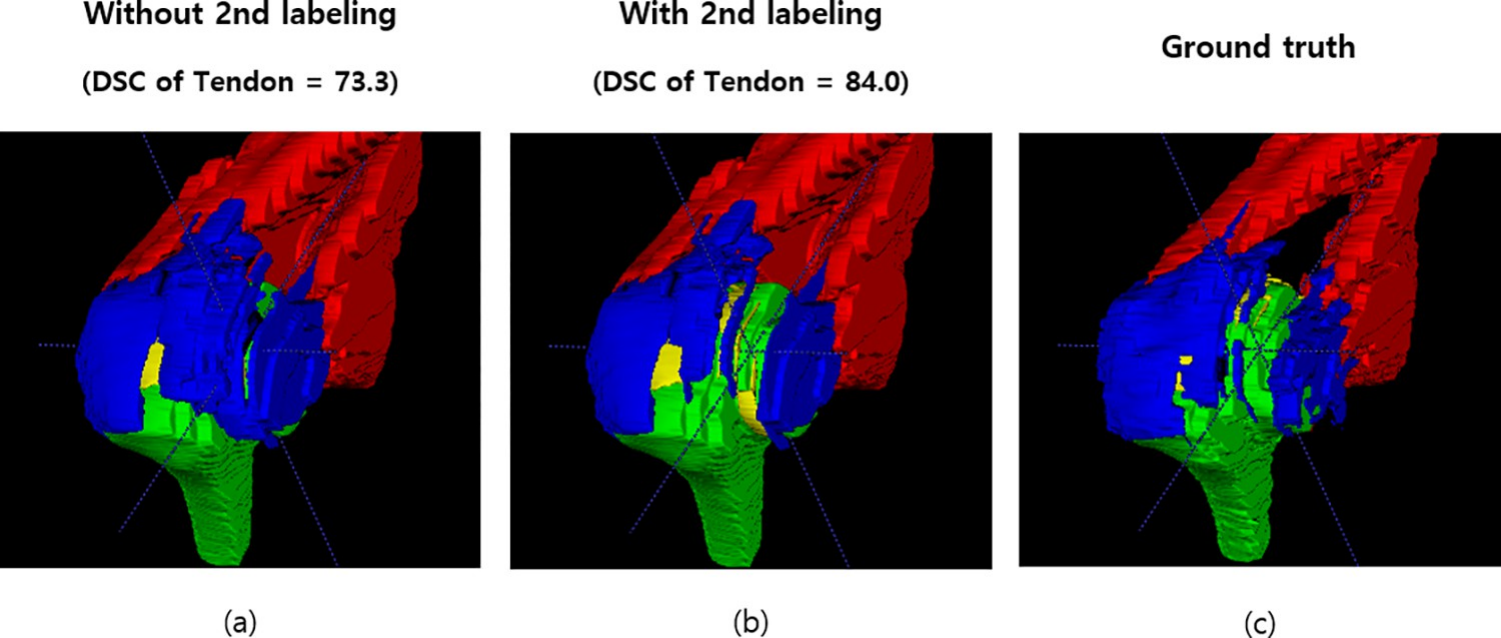
Reduction of false-positive segmentation results using secondary labelling. (a) The result of prediction using 3D U-Net. (b) The result of adapting secondary labelling. (c) Ground truth.

The means and SD of the DSC from the model trained on the test dataset was 0.801 ± 0.097. Except for the humerus, the segmentation results for the muscles, tendons, and cartilage showed DSC values lower than 0.800. The DSC of the tendon was the lowest. Application of secondary labelling reduced false positives and improved the overall segmentation performance (DSC 0.729 → 0.801, p-value = 0.04, Table 2). Fig 6 intuitively shows that false positives are effectively reduced by applying the secondary label in the 3D U-Net. The constructed automated segmentation using 2D and 3D U-Nets required an average inference time of 10 s and 30 s, respectively. For reference, the DSC of intraobserver difference was 0.800 ± 0.103 when the same surgeon drew the tendon cuff again in five cases, while the DSC of interobserver difference was 0.761 ± 0.110.

### Subgroup analysis according to tear size

**Fig 7:**
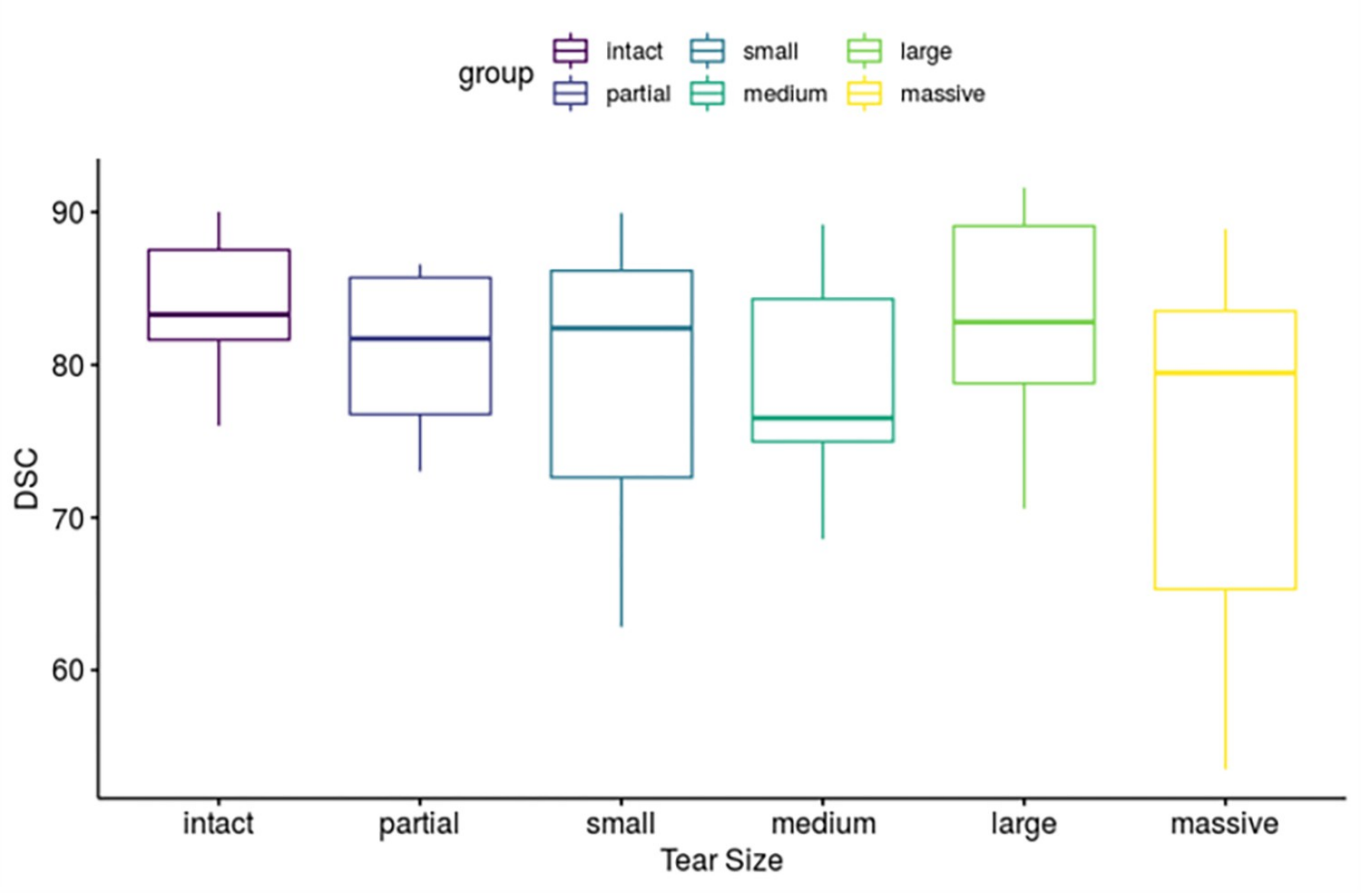
Segmentation performance according to cuff tear size.

Fig 7 shows the results of DSC evaluation based on tear size. In Fig 7, as the x-axis increases to the right, the tear size of the tendon increases, and the y-axis is the DSC performance value of the artificial intelligence model. There was no significant statistical difference in DSC according to tear size among groups.

### External validation results

External validation was performed to confirm if the trained model could be applied to MRI scans performed at outside centers with different protocols. Similarly, DSC with ground truth was evaluated for 10 external validation datasets. The DSC for the tendon in external validation was 0.827 ± 0.052.

## Discussion

The most important finding of our study was that our set protocol based on a deep learning system that combined 3D U-Net with secondary labeling yielded a sufficiently accurate and reproducible DSC (intraobserver, 0.801; interobserver, 0.827) that could help clinicians reliably determine the cuff tendon tear pattern. These were validated in intra- and interobserver assessments. In our segmentation task, BLH was not a region of interest, and the objective was to segment only the cuff tendons. However, BLH of similar intensity was repeatedly captured as a false-positive finding. Therefore, secondary labelling was used to effectively remove false-positive results, and it was to be effective in combination with 3D U-Net. Therefore, we proved that the automatic segmentation of 3D images could produce 3D reconstructed images efficiently and accurately in comparison with manual segmentation.

Previous studies showed the clinical usefulness and evaluation of cuff tendon through segmentation [11, 19, 20]. Some proposed the automatic segmentation method, which can help accurately extract the 3D configuration of the supraspinatus. Additionally, 3D reconstructed supraspinatus images were utilized for evaluating the muscle atrophy changes after rotator cuff repair. Despite the ever-present need and high demand for 3D images, the limitations of 3D imaging were mostly technical. To reconstruct the whole cuff tendon consistently, the protocol was not entirely constructed, and manual segmentation for each case was not easily realized. However, the deep learning used in this study resulted in high reproducibility and accuracy. Moreover, the mean inference time for reconstruction images was taken as only 30 seconds for each case using 3D U-Net. We could overcome many of the limitations using the Deep learning technique, which gave us the direction to move forward.

This study showed that the combination of deep learning with 3D U-Net and Secondary labelling showed the most acceptable results in comparison with the gold standard. In the deep learning process, the actual anatomical structure and its relationships with the surrounding structures were analyzed together to increase the accuracy of the consistent directional result. The deep learning system based on the 2D protocol could only study one slice per segmentation, thereby rendering its accuracy inferior to the 3D protocol, which could determine the relationship with the slices above and below the slice under consideration. Additionally, in the comparison based on the number of variables, no negative linear correlation was observed between the number of variables and accuracy. Rather, the accuracy of tendon evaluation increased when the relationship between the BLH tendon and the cuff tendon was specified. These findings proved that learning the anatomical relationship surrounding the cuff tendon increases reproducibility and accuracy in soft tissue reconstruction.

The clinical value of these findings can be expected to increase since CT images reconstructed as 3D images have shown many clinical applications. Establishment and widespread application of this technology can increase its utilization and research value in the future. Although we have only shown the application of this technique in the visualization of a torn cuff, further research regarding the operation plan depending on the tear pattern and related outcomes might be worth consideration. In addition, by matching the reconstructed images with scope photos, the gold standard of analysis of tear patterns and its accuracy and utilization could be further improved.

The strength of this study is that it confirmed the remarkably increased efficiency and reproducibility of segmentation using deep learning. In addition, those results were obtained from learning various tear models and were validated with other external protocols. These findings suggested that our constructed protocol could yield acceptable results even when applied to different MRI protocols. Nevertheless, this study had several limitations. First, the number of patients was relatively small. Since it takes a lot of time and resources to additionally secure segmented labelling, we plan to proceed further. Second, the overall DSC of the reconstructed 3D images was approximately 0.830. However, since the intra/interobserver segmentation variability for the cuff tendon DSCs was 0.800 and 0.761, respectively, the performance can be considered to be sufficiently high. Moreover, when the size of the segmentation object is small or thin, the DSC value is undoubtedly low. Third, even though we could measure the muscle volume, we could not evaluate the quality of the muscle and tendon.

## Conclusion

Automated segmentation using 3D U-Net produced acceptable accuracy and reproducibility. To overcome the confusion caused by the similar strengths of cartilage and tendons in MRI, we significantly improved segmentation performance by using secondary labelling with the AI model to refine and retrain the data. This method could provide rapid, intuitive visualization that can substantially facilitate diagnosis and treatment planning in patients with rotator cuff tears.
